# *Tinodeslumbardhi* sp. nov. (Trichoptera, Psychomyiidae), a new species from the Lumbardhi i Deçanit River in Kosovo

**DOI:** 10.3897/BDJ.13.e143104

**Published:** 2025-01-20

**Authors:** Halil Ibrahimi, Astrit Bilalli, Milaim Musliu, Donard Geci, Linda Grapci Kotori

**Affiliations:** 1 University of Prishtina "Hasan Prishtina", Faculty of Mathematics and Natural Sciences, Department of Biology, Prishtina, Kosovo University of Prishtina "Hasan Prishtina", Faculty of Mathematics and Natural Sciences, Department of Biology Prishtina Kosovo; 2 Univeristy of Peja "Haxhi Zeka", Faculty of Agribusiness, Pejë, Kosovo Univeristy of Peja "Haxhi Zeka", Faculty of Agribusiness Pejë Kosovo

**Keywords:** Balkan Peninsula, *Tinodesurdhva*, *Tinodeskimminsi*, caddisfly diversity, taxonomy, microscale endemism, anthropogenic pressures

## Abstract

**Background:**

Knowledge about the caddisfly fauna of Kosovo has expanded significantly in recent years; however, new species continue to be described from less-studied regions.

**New information:**

In this paper, we describe a new species, *Tinodeslumbardhi* sp. nov., from the Lumbardhi i Deçanit River in the Bjeshkët e Nemuna Mountains of Kosovo. The new species is closely related to *Tinodesurdhva* Olah, 2010 and *Tinodeskimminsi* Sykora, 1962, but differs in the shapes of segment IX, coxopodites, harpago, phallicata and the basal plate process.

*Tinodeslumbardhi* sp. nov. represents the sixth recorded species of the genus *Tinodes* Curtis, 1834, in Kosovo. The species was found in a small, isolated population within a region highly impacted by anthropogenic activities, especially the construction of hydropower plants. *Tinodeslumbardhi* sp. nov. was found in sympatry with some species rarely encountered in Kosovo, such as *Plectrocnemiageniculata* McLachlan, 1871, *Rhyacophilaloxias* Schmid, 1970 and *Rhyacophilasiparantum* Ibrahimi, Bilalli & Kučinić, 2021. Ongoing human activities have placed increasing pressures on the river’s ecosystem, further emphasising the conservation importance of identifying and protecting these rare and specialised species within Kosovo’s freshwater habitats.

## Introduction

Over the past decade, research on freshwater ecosystems in Kosovo has significantly expanded, covering taxonomy, ecology, biomonitoring and molecular analysis for several groups. The overall assessment outlined from these investigations is that these ecosystems are under continuous anthropogenic pressures, thus endangering the exceptionally diverse fauna (Gashi et al. 2016, Musliu et al. 2018, Grapci-Kotori et al. 2020, Schabetsberger et al. 2020, Ibrahimi et al. 2021b, Bilalli et al. 2022, Buçinca et al. 2024, Zogaris et al. 2024). This progress in investigations has also led to the continuous description of new species of caddisflies from previously underexplored regions. The Bjeshkët e Nemuna Mountains, a crucial biodiversity hotspot in Kosovo, has been the focus of few non-systematic caddisfly studies. Notably, certain caddisfly species are known in Kosovo exclusively from the Bjeshkët e Nemuna Mountains. These include: *Drususkrusniki* Malicky, 1981, *Ernodesskipetarum* Malicky, 1986, *Drususvekon* Ibrahimi & Olah, 2017, *Drususfortos* Ibrahimi & Olah, 2017, *Potamophylaxcoronavirus* Ibrahimi, Bilalli & Vitecek, 2021 and *Rhyacophilasiparantum* Ibrahimi, Bilalli & Kucinic, 2021, the last four species being endemic to Kosovo (Oláh et al. 2017, Ibrahimi et al. 2014, Ibrahimi et al. 2015, Ibrahimi et al. 2019a, Ibrahimi et al. 2019b, Ibrahimi et al. 2021b, Ibrahimi et al. 2021c, Ibrahimi et al. 2021d). Several other endemics are known from the Bjeshkët e Nemuna part of Albania (Oláh 2010, Oláh et al. 2017). The unique species composition highlights the ecological importance of the Bjeshkët e Nemuna Mountains and emphasises the need for continued conservation efforts in these biologically rich habitats under increasing environmental pressures.

The genus *Tinodes*, notable for its diversity, has a broad distribution across Europe, Africa and Asia, with many species exhibiting restricted ranges and often limited to specific, ecologically distinct freshwater habitats (e.g. Malicky 2004, Oláh 2010, Ibrahimi et al. 2018, Ibrahimi et al. 2019c, Cerjanec et al. 2020, Ibrahimi et al. 2021a, Hinić-Jordanovska et al. 2024), highlighting their sensitivity to environmental changes. The ecological specificity of *Tinodes* species makes them particularly valuable in studies of endemism and biodiversity within freshwater systems and in biomonitoring to assess habitat quality and conservation status.

In this paper, we describe a new species of *Tinodes* from the Lumbardhi i Deçanit River in the Bjeshkët e Nemuna Mountains of Kosovo, provide an overview of sympatric species, discuss anthropogenic pressures on these ecosystems and provide a list of species of the Psychomyiidae family in the country.

## Materials and methods

### Fieldwork and laboratory processing

We collected adult caddisflies with ultraviolet light traps, placed nearby the stream. Specimens were stored directly in 80% ethanol. Abdomen of the holotype was macerated in potassium hydroxide (KOH) and kept in glycerine. The rest of the specimen was kept in 70% alcohol. Holotype is deposited in the collection of Halil Ibrahimi, Faculty of Mathematics and Natural Sciences, University of Prishtina “Hasan Prishtina”, collection name “Deçan”.

Morphological features of genitalia of *Tinodeslumbardhi* sp. nov. were analysed from two male specimens. For comparative assessments of morphological features, we used specimens of *Tinodeskimminsi* collected in North Macedonia (Musliu et al. 2020, Hinić-Jordanovska et al. 2024) and *Tinodesurdhva* collected in Albania (Oláh 2010).

Illustrations were prepared in Adobe Illustrator (version Creative Cloud 2018) by digitising pencil templates made with an Olympus S50 camera. Systematic nomenclature follows Morse (2024). The description terminology follows Nielsen (1957) with the exception of 'intermediate appendage' used instead of 'paraproct'. 'Process of basal plate' is used according to Oláh (2010) for 'phallic guide'.

### Sampling area

**Figure 1.**
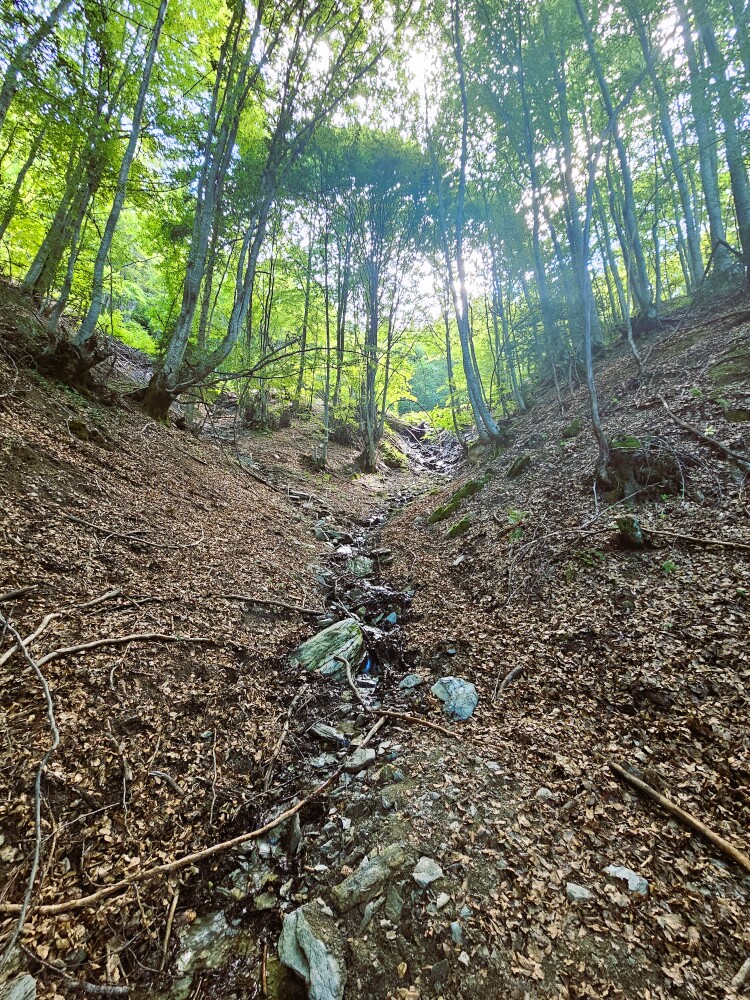
Type locality of *Tinodeslumbardhi* sp. nov. in the Bellaja Village.

The sampling site is located in a small stream in the Village of Bellaja, within Deçan Municipality, part of the Bjeshkët e Nemuna Mountains in western Kosovo (Fig. 1). This stream is a tributary of the Lumbardhi i Deçanit River. Bjeshkët e Nemuna is a prominent mountain range on the Balkan Peninsula, extending from northern Albania through western Kosovo to north-eastern Montenegro. The range is characterised by its high peaks and rugged terrain, including Maja e Jezercës, which stands at 2,694 m. This is at the same time the highest peak in the entire Dinaric Alps. Within Kosovo, the range features Gjeravica Mountain, which, at 2,656 m, is the second-highest peak in the country.

The Lumbardhi i Deçanit River originates from the southern slopes of the Bogiçevica Mountain and flows northwards past the Gjeravica peak. Initially known as Lumi Shqiptar in its upper reaches, this river gathers waters from several smaller streams descending from nearby mountains. It discharges into the Drini i Bardhë River near Peja Town and belongs to the Adriatic Sea Basin.

## Taxon treatments

### Tinodes lumbardhi

Ibrahimi, Bilalli & Musliu sp. nov.

4E36CB39-A49F-5545-A950-679D77193272

#### Materials

- **Type status:** Holotype. **Occurrence:** lifeStage: adult; occurrenceID: B744309F-37E6-59B7-BAE5-688AC4CFD788; **Location:** continent: Europe; waterBody: Adriatic Sea Basin; country: Kosovo; countryCode: XK; municipality: Deçan; locality: Bellaja Village, sidestream of the Lumbardhi i Deçanit River; verbatimCoordinates: 42.581919°N, 20.200688°E; decimalLatitude: 42.581919; decimalLongitude: 20.200688°; **Event:** samplingProtocol: UV light trap; samplingEffort: 2 trap-nights; eventDate: 2024-06-21; year: 2024; month: 06; day: 21
- **Type status:** Paratype. **Occurrence:** lifeStage: adult; occurrenceID: DD86C4A1-5EBF-5411-AEBF-11B01B56B0C5; **Location:** continent: Europe; waterBody: Adriatic Sea Basin; country: Kosovo; countryCode: XK; municipality: Deçan; locality: Bellaja Village, sidestream of the Lumbardhi i Deçanit River; verbatimCoordinates: 42.581919°N, 20.200688°E; decimalLatitude: 42.581919; decimalLongitude: 20.200688; **Event:** samplingProtocol: UV light trap; samplingEffort: 2 trap-nights; eventDate: 2024-06-21; year: 2024; month: 06; day: 21

#### Description

Male (in alcohol). Forewing length 4.7-4.9 mm; head light brown, antennae brown, maxillary palps brown; thorax light brown; legs yellow-brown, number of spurs 2, 4, 4; abdomen dark brown, external genital apparatus light brown to brown.

Genitalia (Fig. 2). Sternite IX with longer dorsal rod-shaped portion and square-like ventral portion, proximal margin straight, basally rounded, apex of the ventral part of sternite IX slightly widening, in lateral view. In ventral view, segment IX roughly quadratic in shape. Tergite IX sclerotised dorsally, membranous ventrally. Superior appendages long, thick, sinuate, dilating medially, not reaching the apex of inferior appendages, tapering apically and bearing long setea on the surface. Intermediate appendages consist of a pair of broad dorsal processes, shorter than superior appendages, that nearly encase the phallic organ in a tubular fashion. Each intermediate appendage is equipped with megasetae positioned within well-developed alveoli. Coxopodites of inferior appendages relatively long in lateral view, highest mesally, with convex dorsal and ventral margins and concave proximal margin; in ventral view, fused mesoventrally near the base, inner margin wide and V-shaped. Harpagones simple monolobed, broader basally, apically rounded and turned mesad in lateral view, equally broad and apically truncated, slightly turned inwards in ventral view. The phallic apparatus is positioned within the intermediate appendage processes, apically encircled by a membranous process.

#### Diagnosis

Males of *Tinodeslumbardhi* sp. nov. are most similar to *Tinodeskimminsi* and *Tinodesurdhva*, but differ in the shapes of segment IX, the coxopodites, harpagones, phallicata and the basal plate process.

In *Tinodeslumbardhi* sp. nov., segment IX is proximally straight, whereas it is convex in both *Tinodeskimminsi* and *Tinodesurdhva*.

The coxopodites of *Tinodeslumbardhi* sp. nov. are large and roughly rectangular, with a broad, angled apex in lateral view, unlike those of *Tinodesurdhva* and *Tinodeskimminsi*, which are oviform with an acuminate apex. The harpagones of *Tinodeslumbardhi* sp. nov. are large, basally wide, apically rounded and mesad-turned in lateral view, appearing equally wide and apically truncated in ventral view. In contrast, in *Tinodesurdhva*, the harpagones are small, upturned in lateral view, with a very wide base and an acuminate apex in ventral view. In *Tinodeskimminsi*, the harpagones are large and evenly wide in lateral view, slightly turned downwards, with an apically truncated shape in lateral view; they appear almost straight and apically truncated, in ventral view.

The apex of the basal plate of the inferior appendages in *Tinodeslumbardhi* sp. nov. is small, ventrally rounded in lateral view, while, in *Tinodeskimminsi*, it is large and with a single long pointed ventral process and, in *Tinodesurdhva*, it is large and has two small ventral pointed processes.

The megasetae of intermediate appendages in *Tinodeslumbardhi* sp. nov. are arranged as follows: one middle ventral turned upwards, two subapical ventral, one very small seta located between the middle ventral and subapical setae, four apical, one long middle dorsal and six subapical dorsal setae. In *Tinodeskimminsi*, there is one middle ventral seta turned upwards, three subapical ventral and three middle dorsal setae; in *Tinodesurdhva*, there is one middle ventral seta turned downwards, with 2–3 subapical ventral, 3–4 apical and 5–6 subapical dorsal setae.

In *Tinodeslumbardhi* sp. nov., the phallicata is a simple rod surrounded by a less sclerotised membranous structure and an additional smaller apical membranous structure. In *Tinodeskimminsi*, the phallicata is a simple rod encircled by a very broad, less sclerotised membranous structure. In *Tinodesurdhva*, the phallicata is a simple rod without any surrounding structures.

In addition, *Tinodeslumbardhi* sp. nov. differs from *Tinodesurdhva* by having longer and more slender coxopodites in ventral view, as well as a deeper incision. The median ventral processes of the basal plate of the gonopods are also shaped differently between the two species in lateral view. Furthermore, the basal part of the basal plate of the gonopods in *Tinodeslumbardhi* sp. nov. is wider than in *Tinodesurdhva* in ventral view.

#### Etymology

This species is named after the river where it was discovered. The species epithet, *lumbardhi*, is a noun in apposition derived from the Albanian name of the river, meaning 'the white river.' As a noun in apposition, it does not need to conform to Latin grammatical gender.

#### Distribution

Kosovo: Lumbardhi i Deçanit River, Bjeshkët e Nemuna.

#### Ecology

The species was found in a small, shaded stream with minimal water flow. The stream was surrounded by dense, high vegetation that provides near-constant shade, creating a cool and dimly lit environment. The substrate was composed of small rocks, pebbles, sand and a substantial accumulation of fallen leaves and branches, which contribute to a rich layer of organic material on the streambed. This shaded, leaf-littered environment likely plays a significant role in the species’ microhabitat preferences. The species was found in sympatry with the following species: *Rhyacophilaloxias*, *Rhyacophilasiparantum*, *Rhyacophilatristis* Pictet, 1834, *Philopotamusmontanus* Donovan, 1813, *Polycentropusirroratus* Curtis, 1835, *Plectrocnemiaconspersa* Curtis, 1834, *Plectrocnemiageniculata* McLachlah, 1871, *Tinodesrostocki* McLahlan, 1878 and *Sericostomaflavicorne* Schneider, 1845.

## Discussion

Kosovo and the broader area of the Balkan Peninsula provide favourable conditions for a rich caddisfly fauna within Europe and the Western Palearctic Region (Ibrahimi 2024). The region's diverse caddisfly communities are rooted in its complex evolutionary past and a rich array of aquatic habitats, which have fostered high species diversity and unique endemism. However, aquatic insects, including caddisflies, face mounting environmental pressures that jeopardise their populations and habitats. Human-driven impacts in Kosovo, such as the development of hydropower plants, pollution and extensive water extraction, pose significant risks to freshwater ecosystems and can lead to local extinctions.

*Tinodeslumbardhi* sp. nov. belongs to the *Tinodesunicolor* species group, a group noted for its restricted distribution. The discovery of *Tinodeslumbardhi* sp. nov. adds to Kosovo’s biodiversity catalogue and emphasises the region’s importance as a biodiversity hotspot for caddisflies.*Tinodeslumbardhi* sp. nov. is the sixth known species of the genus *Tinodes* from Kosovo (Ibrahimi et al. 2012a, Ibrahimi et al. 2014, Ibrahimi et al. 2015a). With this addition, the family Psychomyiidae is now represented in Kosovo with ten species (Table 1). Some species of the Psychomyiidae family are relatively rare in Kosovo. For example *Lypereducta* Stephens, 1836 was reported from a single locality in Kosovo more than a century ago (Ibrahimi et al. 2012a) and has never been recorded since that time. *Psychomyiaklapaleki* Malicky, 1995 and *Tinodesbraueri* McLachlan, 1878 are reported each from a single locality in Kosovo as well (Ibrahimi et al. 2015).

**Figure 3.**
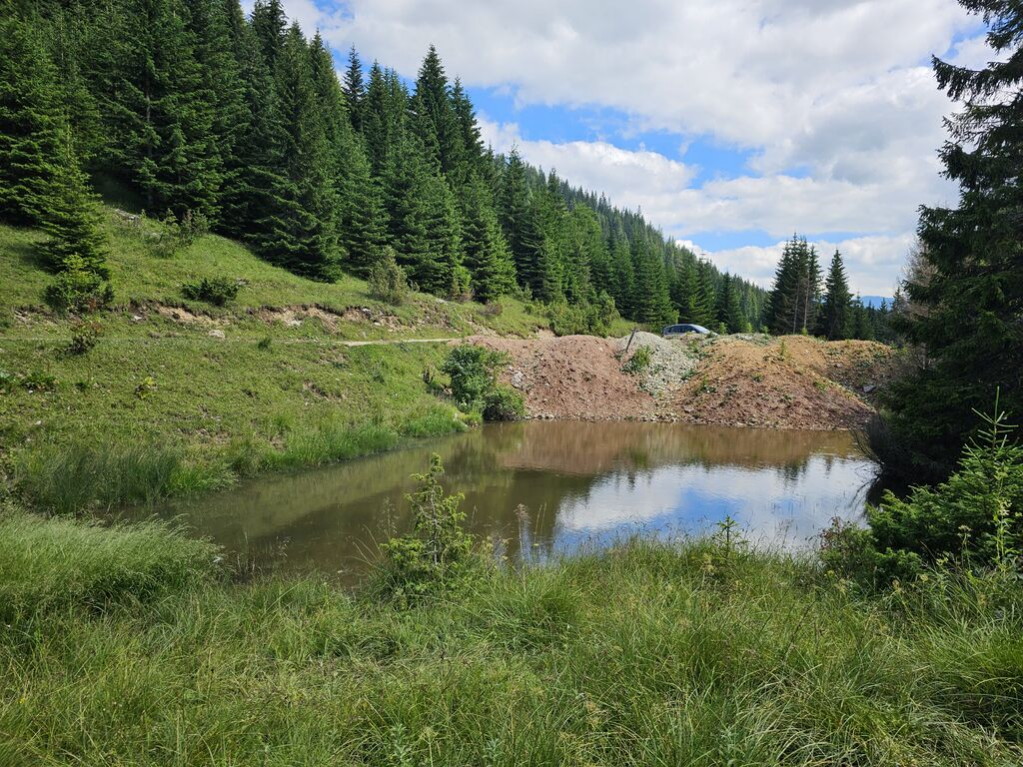
The deteriorated type locality of *Rhyacophilasiparantum* at the Bogë Village in Bjeshkët e Nemuna. Photo taken on 21 June 2024.

*Tinodeslumbardhi* sp. nov. was discovered in sympatry with several rarely encountered species in Kosovo (Ibrahimi et al. 2019a, Ibrahimi et al. 2021d, Bilalli et al. 2024), including *Plectrocnemiageniculata*, *Rhyacophilaloxias* and *Rhyacophilasiparantum*. Notably, the presence of *Rhyacophilasiparantum* alongside the newly-described species is of particular significance, given recent concerns about its habitat at the type locality. *Rhyacophilasiparantum*, a species previously known only from the type locality (Ibrahimi et al. 2021d, Bilalli et al. 2024), was not recorded during 2024 surveys at this site despite extensive sampling efforts. This absence is likely attributable to habitat degradation, as the stream flow has been disrupted by a large accumulation of soil resulting from nearby road construction (Fig. 3). The alteration has created a pond-like environment, which appears to have significantly affected the caddisfly community within the stream, including the apparent disappearance of *Rhyacophilasiparantum*.

The Bellaja Stream, where the new species was discovered, is a side-stream of the Lumbardhi i Deçanit River, a watercourse that has experienced significant anthropogenic pressures in recent years (Fig. 4). Preliminary results from a recent study (Ibrahimi, unpublished data) reveal that the main reach of the Lumbardhi i Deçanit River is highly degraded, primarily due to the construction and operation of hydropower plants. These activities have led to impoverished aquatic insect communities along the river’s main flow and a complete absence of fish in several segments within a wide radius of hydropower plant influence. Consequently, rare species of caddisflies and other aquatic insects persist only in isolated patches of sidestreams within the Lumbardhi i Deçanit watershed.

These findings underscore the urgent need for targeted conservation measures to mitigate the impacts of anthropogenic activities on freshwater habitats and their biodiversity. The preservation of these rare caddisfly species is crucial not only for maintaining the biodiversity of Kosovo’s freshwater ecosystems, but also for safeguarding their ecological stability. Caddisflies are well-documented bioindicators, with many species exhibiting high sensitivity to pollution and habitat alteration. Their presence and survival in these sidestream refuges signals the critical importance of protecting such microhabitats.

Moreover, the discovery of new species in Kosovo emphasises the necessity of proactive conservation strategies focusing on streams and other aquatic habitats. Addressing the multifaceted threats posed by hydropower development and other anthropogenic activities is imperative for preserving the complex interactions between these rare species and their habitats (Ibrahimi et al. 2024).

## Supplementary Material

XML Treatment for Tinodes lumbardhi

## Figures and Tables

**Figure 2a.**
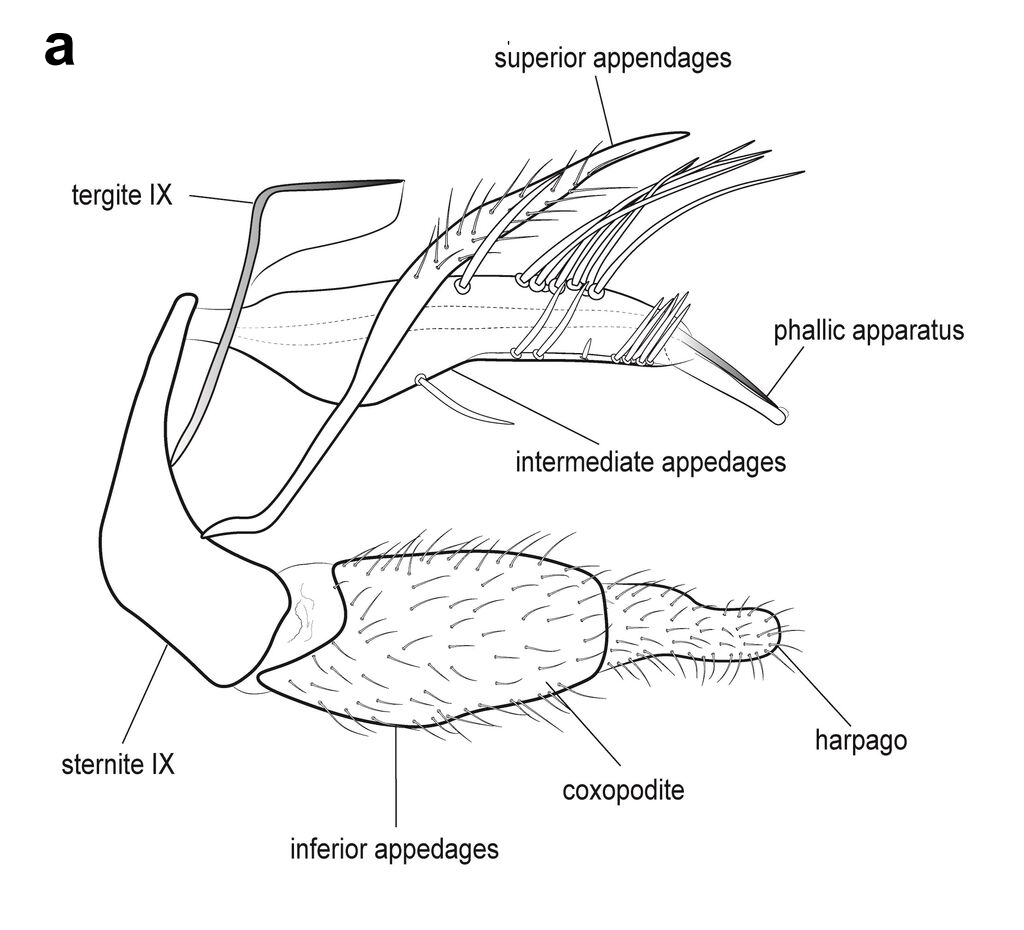
Lateral view;

**Figure 2b.**
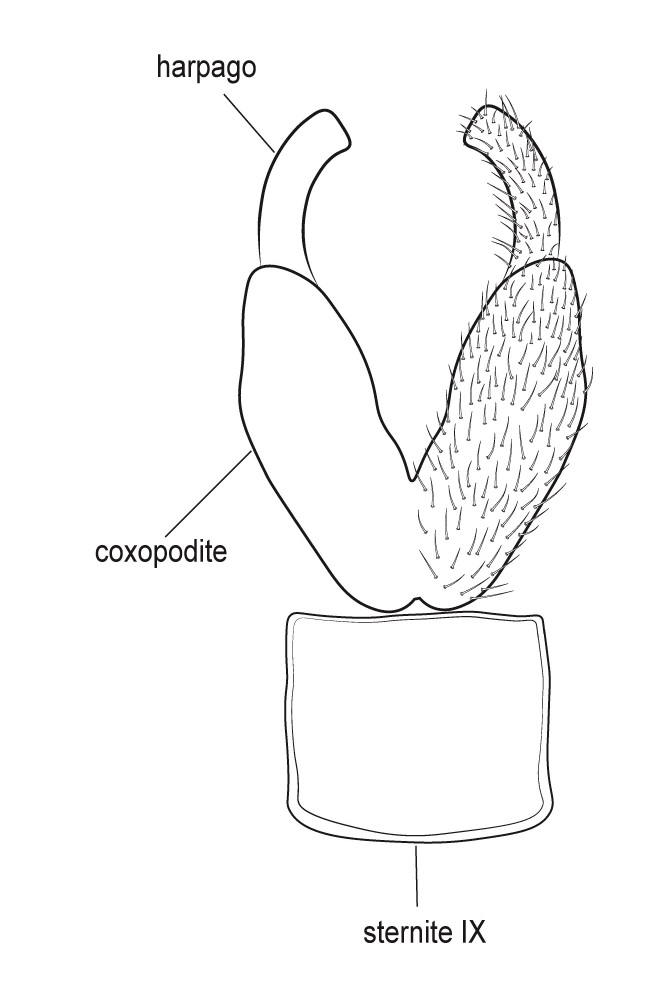
Ventral view;

**Figure 2c.**
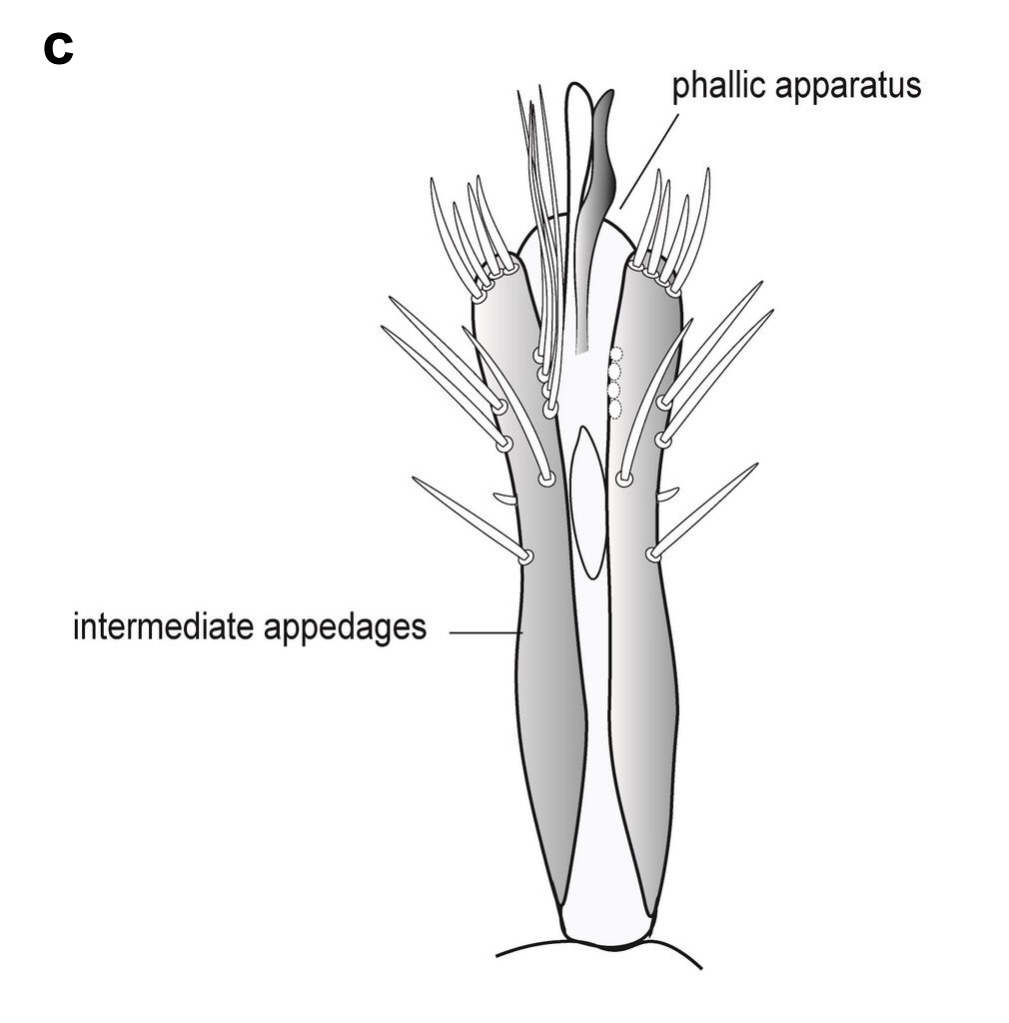
Phallus and intermediate appendages, dorsal view;

**Figure 2d.**
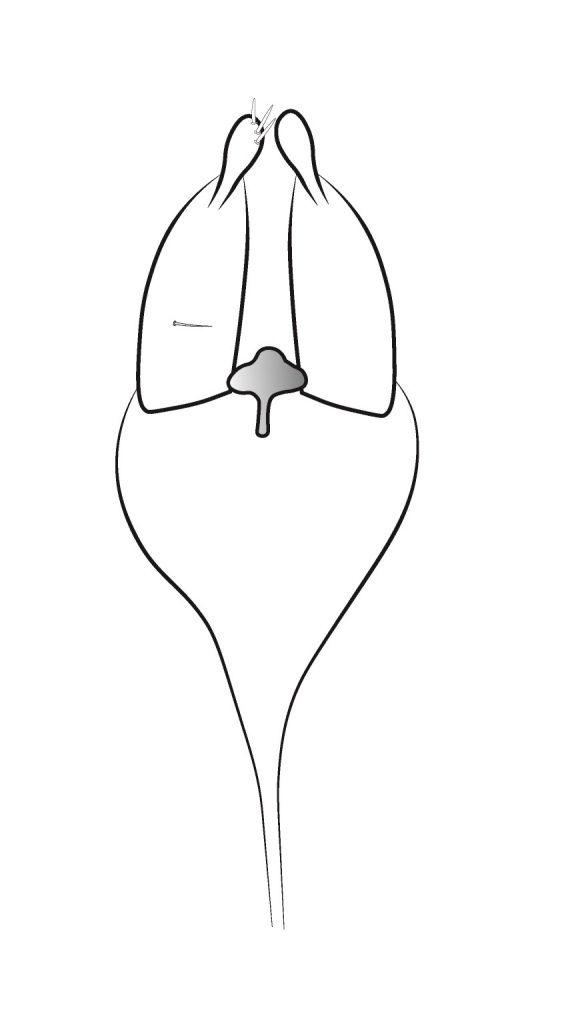
Process of basal plate, ventral view;

**Figure 2e.**
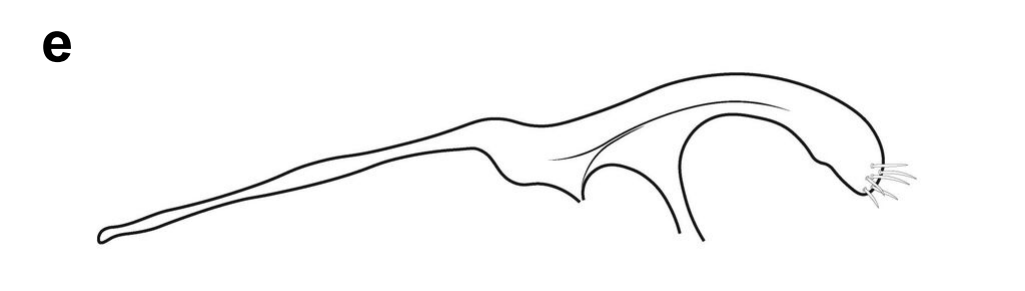
Process of basal plate, lateral view.

**Figure 4a.**
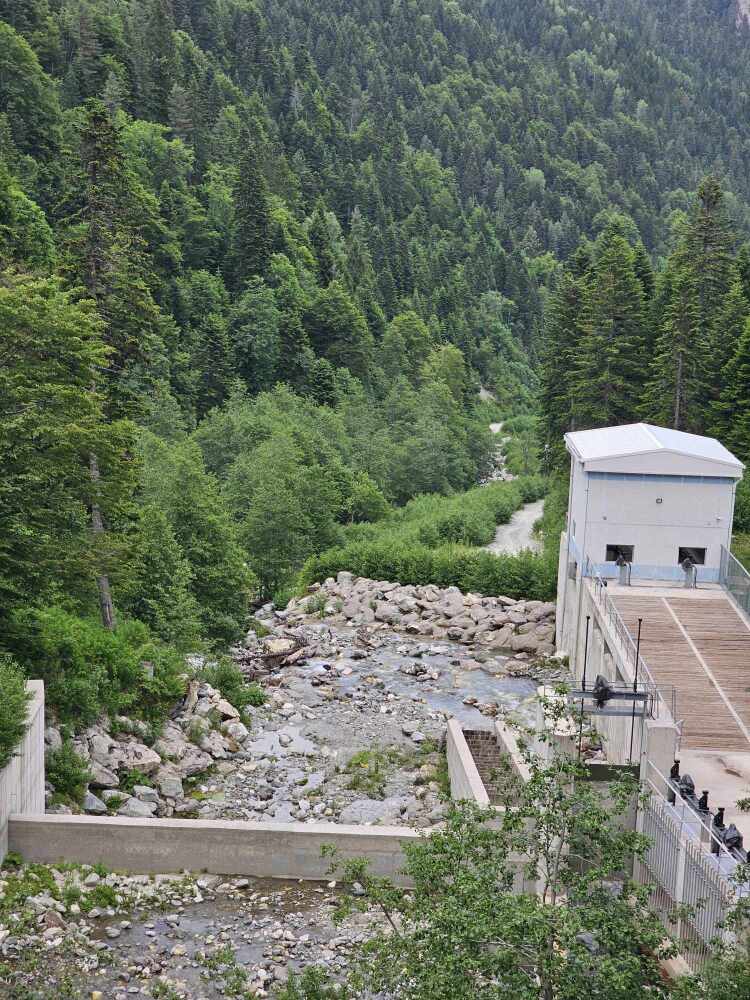
Hydropower plant pressure, nearby the type locality of *Tinodeslumbardhi* sp. nov.;

**Figure 4b.**
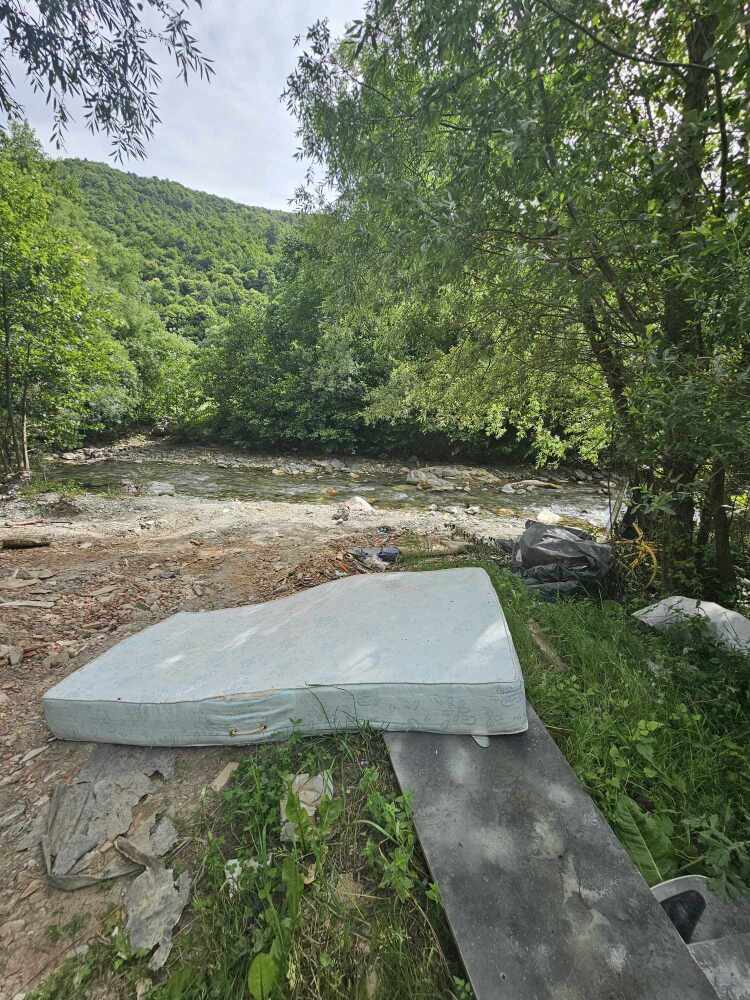
Garbage along the Lumbardhi i Deçanit River;

**Figure 4c.**
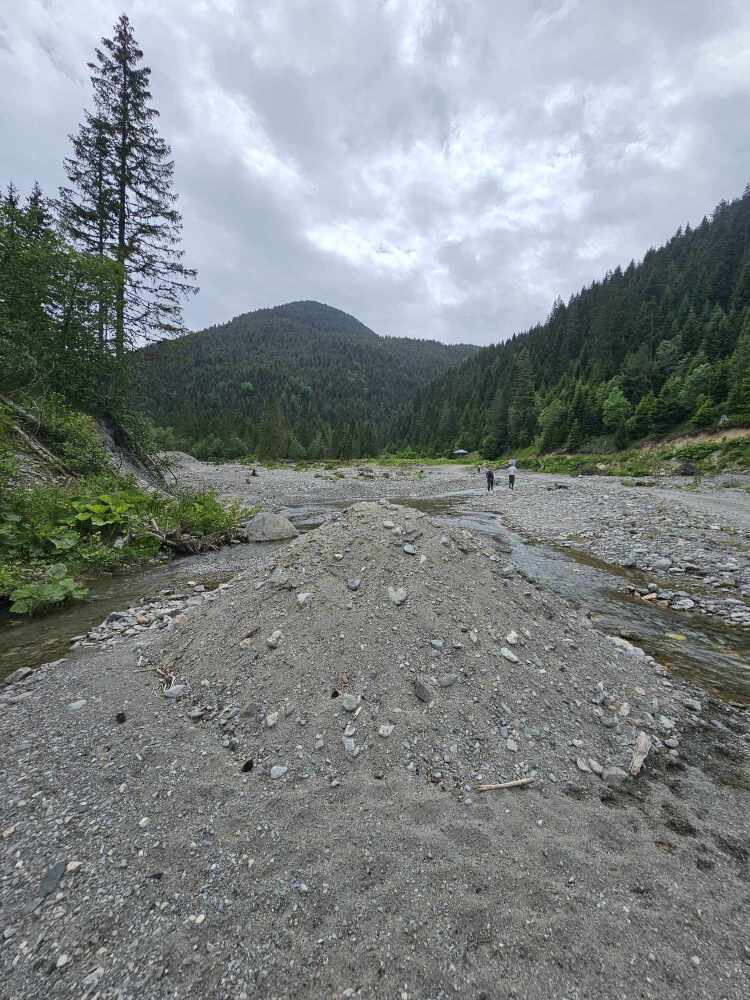
Alteration of riverbed at the upstream area of the Lumbardhi i Deçanit River, Zalli i Rupës;

**Figure 4d.**
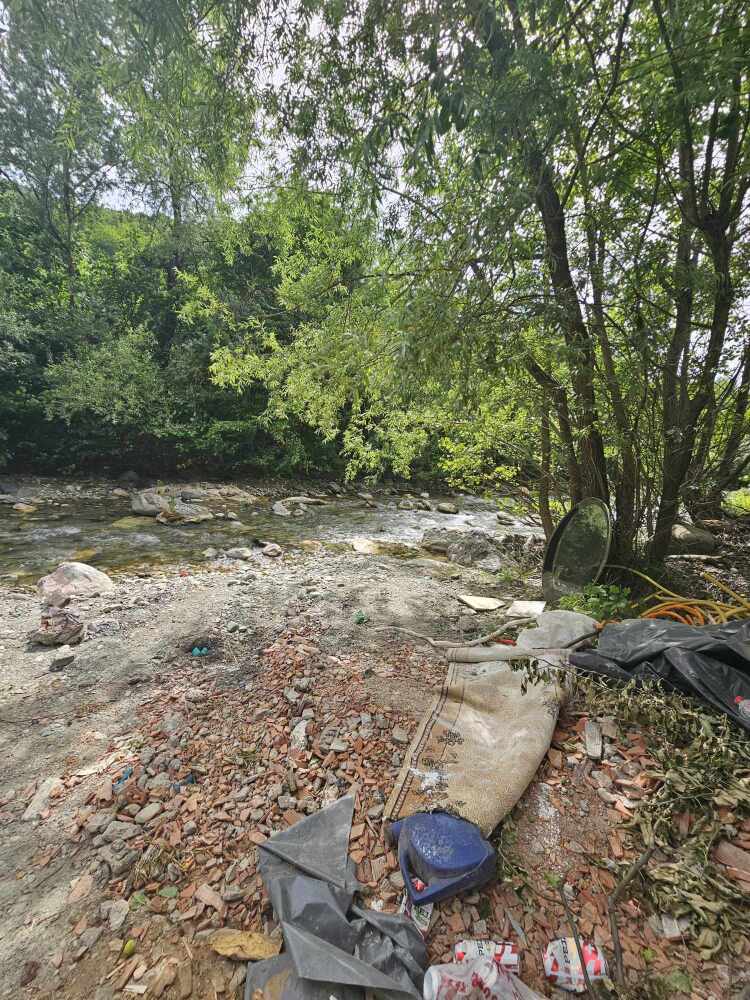
Garbage along the Lumbardhi i Deçanit River;

**Figure 4e.**
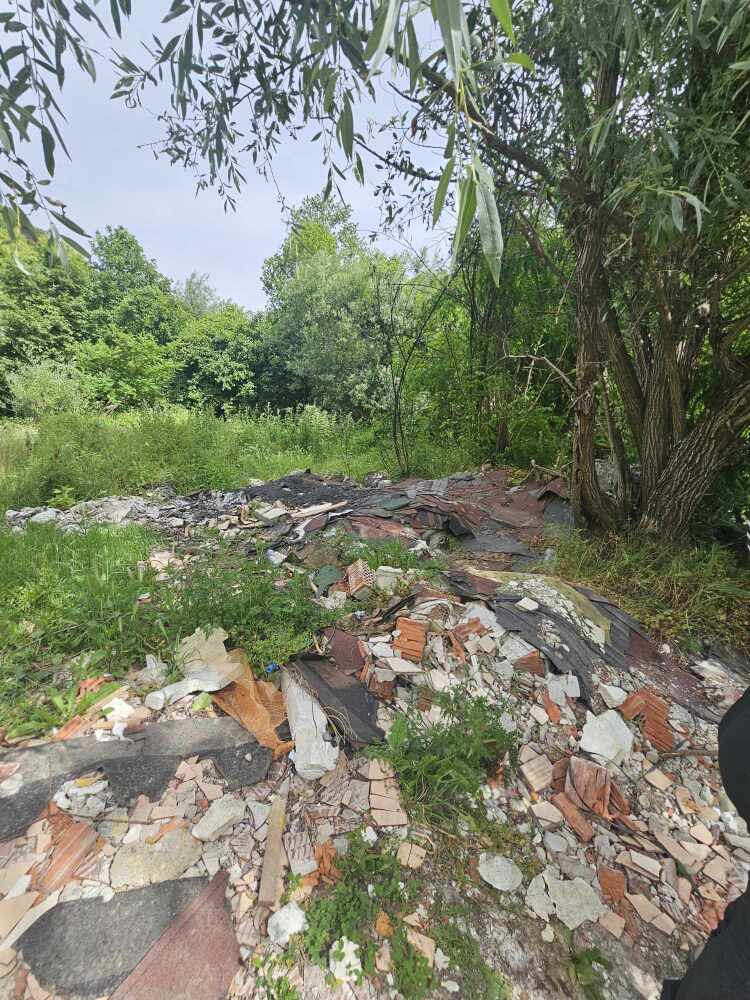
Garbage along the Lumbardhi i Deçanit River.

**Table 1.** The list of species of the Psychomyiidae family in Kosovo with distribution details.

**Species**	**Distribution**	**References**
*Lypephaeopa* Stephens, 1836	Pejë	Pongrácz (1923)
*Lypereducta* Hagen, 1868	Blinajë; Sredskë, Kuqishte; Mirushë, Bajgorë; Llap; Gollak	Ibrahimi et al. (2012b), Ibrahimi et al. (2014), Salihu et al. (2023)
*Psychomyiaklapaleki* Malicky, 1995	Ibër River	Ibrahimi et al. (2012a)
*Psychomyiapusilla* Fabricius, 1781	Widespread in Kosovo	Ibrahimi et al. (2012a), Ibrahimi et al. (2014), Ibrahimi et al. (2019c), Salihu et al. (2023), Musliu et al. (2024)
*Tinodesbraueri* McLachlan, 1878	Side-stream of Erenik River	Ibrahimi et al. (2015)
*Tinodesjanssensi* Jacquemart, 1957	Blinajë; Lipjan; Dërmjak	Ibrahimi et al. (2012a), Ibrahimi et al. (2016), Musliu et al. (2024)
*Tinodesrostocki* McLachlan, 1878	Relatively widespread in Kosovo	Ibrahimi et al. (2012a), Ibrahimi et al. (2014)
*Tinodeslumbardhi* Ibrahimi, Bilalli & Musliu sp. nov.	Side-stream of the Lumbardhi i Deçanit River	This paper.
*Tinodespallidulus* McLachlan, 1878	Pejë; Klinë	Ibrahimi et al. (2014)
*Tinodesunicolor* Pictet, 1834	Mollopolc; Vrellë	Ibrahimi et al. (2012a), Ibrahimi et al. (2014)
